# Label-free LC–MS/MS proteomics analyses reveal proteomic changes in oxidative stress and the SOD antioxidant strategy in TM cells

**DOI:** 10.1186/s12014-022-09350-4

**Published:** 2022-05-14

**Authors:** Qian Li, Liyu Zhang, Yuxin Xu

**Affiliations:** Department of Ophthalmology, The Second Affiliated Hospital of Anhui Medical University, Anhui, 230601 China

**Keywords:** Trabecular meshwork cells, Oxidative stress, Label-free, Proteomic analysis, SOD

## Abstract

**Background:**

Treatment for glaucoma has traditionally been limited to reducing intraocular pressure (IOP). Inhibiting oxidative stress in the trabecular meshwork (TM) is regarded as a new treatment for glaucoma; however, the effects do not meet expectations. Exploring the mechanism by which oxidative stress and antioxidant stress occur in TM cells will offer clues to aid the development of new treatments.

**Methods and results:**

In our study, we cultured TM cells and used H_2_O_2_ and SOD to induce and inhibit oxidative stress, respectively. Label-free LC–MS/MS quantitative proteomic analysis was conducted to analyze the differentially expressed proteins and relevant signaling pathways. A total of 24 upregulated proteins and 18 downregulated proteins were identified under oxidative stress. PTGS2, TGFβr2 and ICAM-1 are the key proteins. The PTGS2/NF-ĸb pathway, TGF-β/Smad signaling pathway and AGE-RAGE signaling pathway in diabetic complications may be the major signaling pathways under conditions of ROS-induced damage in TM cells. Seventy-eight proteins were upregulated and 73 proteins were downregulated under antioxidant stress in TM cells. The key protein was ICAM-1, which participates in the African trypanosomiasis pathway, one of the most important pathways under antioxidant stress. Combining the results of the Venn diagram with protein–protein interactions (PPIs), ICAM-1 was identified as the major protein. Cell Counting Kit-8 (CCK-8) and western blotting (WB) were used to reveal that suppressing the expression of ICAM-1 would improve the survival of TM cells.

**Conclusions:**

Key proteins and signaling pathways play important roles in the mechanisms of oxidative stress and antioxidant strategies in TM cells. ICAM-1 knockdown can suppress the apoptosis of TM cells induced by H_2_O_2,_ which may reveal new therapeutic targets and biomarkers for glaucoma.

## Introduction

Glaucoma is a multi‐factorial optic neuropathy characterized by irreversible vision loss and corresponding atrophy of the optic nerve. It has been estimated to affect 3.5% of individuals over 40 years old and is projected to affect a total of 112 million people by 2040 [1]. Although the pathogenesis of glaucoma is not fully understood, the trabecular meshwork (TM) is regarded as the key factor for the worsening of glaucoma. TM can reduce aqueous humor outflow, leading to a high-tension glaucoma cascade [2]. Moreover, it has been hypothesized that glaucomatous TM cells (TMCs) may have altered gene and protein expression, generating molecular signals and contributing to retinal ganglion cell (RGC) death in both normal-tension and high-tension glaucoma [3]. Therefore, understanding the mechanisms of pathological changes in the TM is critical for the prevention and treatment of glaucoma.

Oxidative stress (OS) plays an important role in the pathological mechanism of glaucoma [4], as the associated increases in reactive oxygen species (ROS) can damage the cornea, iris, and TM. Thus, focusing solely on reducing intraocular pressure (IOP) for glaucoma therapy, especially for some patients who do not respond to this type of treatment, is insufficient [5]. Notably, reducing OS is emerging as a therapeutic strategy for glaucoma [6]. Some papers have reported the use of antioxidant drug treatment or OS inhibition for the treatment of glaucoma; however, the effects have not met expectations [7, 8] because the underlying molecular mechanisms are far from elucidated. Izzotti et al. [9] reported that the TM is the most sensitive tissue in the eye. When OS occurs, TM mitochondrial dysfunction, inflammatory cytokine release, and impairment of extracellular matrix (ECM) components may occur [4, 10]. Therefore, exploration of how ROS damage the TM and exactly how OS can be suppressed is crucial for further investigating treatments for glaucoma.

Proteins are major agents in the execution of various physiological and pathological processes. When OS occurs, some specific proteins produced by TMCs may participate in the pathological pathway, causing the glaucoma cascade. Therefore, detecting and quantifying the proteins that are changed in TMCs under OS will offer evidence to elucidate the pathogenesis. Thus far, label-free methods have been widely used in clinical research, biomarker discovery, and personalized medicine [11]. Compared with other proteomics technologies, these methods are more convenient, versatile, and flexible [12]. In this study, we cultured human TMCs (HTMCs) and used H_2_O_2_ to induce OS. We also added superoxide dismutase (SOD), the first-line antioxidant, to inhibit OS in HTMCs. Furthermore, we used label-free techniques to detect differentially expressed proteins (DEPs) in different groups. Through bioinformatics analysis, we sought to identify the key proteins and their related signaling pathways to explore the mechanisms in TMCs under OS or antioxidant stress.

## Materials and methods

### Cell culture and identification

Primary explant-derived HTMCs (iCell Bioscience Inc., China) were grown in DMEM/F12 with 20% serum and kept at 37 °C in a 5% CO_2_ environment. Only cells in the 3rd to 5th passages were used. To identify TMCs, collagen type IV, laminin and fibronectin antigens were detected by immunocytochemistry.

### Sample collection and protein preparation

TMCs were pretreated with SOD (0, 1, 5, or 10 U/mL) for 30 min and then exposed for 24 h to a range of H_2_O_2_ concentrations (0, 50, 100, 150, and 200 μM). The concentrations of H_2_O_2_ and SOD in the group with the most obvious protective effect of SOD were screened through a Cell Counting Kit-8 (CCK-8) assay. The cells were divided into three groups: the control group (without any interference, group C), the H_2_O_2_-treated group (with exposure to H_2_O_2_ only, group H), and the SOD pretreatment group (with exposure to SOD before H_2_O_2_ addition, group S). The cells were collected and stored at − 80 °C after homogenization and centrifugation. Then, a mammalian tissue total protein extraction kit (AP0601-50) was used to extract proteins from the different groups. The protein concentrations were estimated using a Bradford assay kit. Next, 20 µg of protein from each sample was mixed with 5× loading buffer at a ratio of 5:1 (v/v). The supernatant was collected after 5 min of boiling in a water bath and 10 min of centrifugation at 14,000×*g*. The proteins were separated on a 10% SDS-PAGE gel (constant current of 14 mA, 90 min). Coomassie Blue R-250 staining was used to visualize the protein bands. After quantification, two hundred micrograms of protein from each sample was incorporated into 30 μL SDT buffer (4% SDS, 100 mM DTT, 150 mM Tris–HCl pH 8.0) and incubated at 37 °C for 1 h. DTT and other low-molecular-weight components were removed using UA buffer (8 M urea, 150 mM Tris–HCl pH 8.5) by repeated ultrafiltration (Sartorius, 10 kD). Then, 100 μL iodoacetamide (100 mM IAA in UA buffer) was added to block reduced cysteine residues, and the samples were incubated for 1 h in darkness. After that, we added 100 μL of NH_4_HCO_3_ (50 mM) to dilute the UA, mixed the samples and centrifuged them under the same conditions. The supernatant was removed as before; this step was repeated three times. Finally, we replaced the collection tube with a new collection tube, added trypsin at a ratio of 50:1 (protein:trypsin) to the digested proteins and incubated the samples at 37 °C for 16 h. After digestion, the peptides were vacuum dried, dissolved in 0.1% trifluoroacetic acid (TFA), desalted on C18 cartridges (Empore SPE Cartridges C18, standard density), dried by vacuum centrifugation and reconstituted in formic acid (FA).

### LC–MS/MS analysis

The reconstituted peptides were analyzed with a Q-Exactive mass spectrometer (Thermo Fisher Scientific, Waltham, MA, USA) coupled with a nano high-performance liquid chromatography (UltiMate 3000 LC Dionex; Thermo Fisher Scientific) system. After protease hydrolysis, 800 ng of proteins from different samples were pressure-loaded onto a C18-reversed-phase column (3 μm-C18 resin, 75 μm × 15 cm) and separated on an analytical column (5 μm C18 resin, 150 μm × 2 cm) using mobile phases A: 0.5% formic acid [FA]/H_2_O and B: 0.5% FA/ACN at a flow rate of 300 nL/min. The chromatographic separation gradient is shown in Table 1. Spectra were acquired in data-dependent mode. The 10 most intense ions selected for MS scanning (300–1800 m/z, 60,000 resolution at m/z 400, accumulation of 1 × 106 ions for a maximum of 500 ms, 1 microscan). The isolation window was 1.3 m/z, and the MS/MS spectra were accumulated for 150 ms using an Orbitrap. MS/MS spectra were measured at a resolution of 15,000 at m/z 400. Dynamic precursor exclusion was allowed for 120 s after each MS/MS spectrum measurement and was set to 17,500 at m/z 200. The normalized collision energy was 30 eV, and the underfill ratio, which specifies the minimum percentage of the target value likely to be reached at the maximum fill time, was defined as 0.1%. The instrument was run with peptide recognition mode enabled.

**Table 1:** Chromatographic separation gradient for LC–MS/MS analysis

Time	0	11	48	68	69	75
A%	93%	85%	75%	60%	0%	0%
B%	7%	15%	25%	40%	100%	100%

### Data analysis and bioinformatics analysis

MaxQuant (1.6.17) was used to search the reviewed FASTA database in UniProt with *Homo sapiens* as the organism. The following options were used to identify the proteins: peptide mass tolerance = ± 15 ppm, MS/MS tolerance = 0.02 Da, enzyme = trypsin, missed cleavage = 2, fixed modification: carbamidomethyl (C), variable modification: oxidation (M), database pattern = decoy. The false discovery rate (FDR) for peptides and proteins was set to 0.01. The protein expression data are presented in a heatmap. The DEPs between groups were defined as significantly upregulated or downregulated on the basis of a fold change (FC) ≥ 1.5 and *P* value < 0.05 (upregulated) or a FC ≤ 0.667 and *P* value < 0.05 (downregulated) (experimental group/control group). We used Metascape, a web-based resource (http://metascape.org), to conduct Gene Ontology (GO) analysis and used the Kyoto Encyclopedia of Genes and Genomes (KEGG) Orthology-Based Annotation System (KOBAS) online analysis tool (http://kobas.cbi.pku.edu.cn/) to perform KEGG pathway analyses. Database enrichment analysis was performed using the UniProtKB database (Release 2016 10). GO enrichment included three ontologies (biological process (BP), molecular function (MF), and cellular component (CC)). In addition, we performed protein–protein interaction (PPI) analysis using STRING software (http://string-db.org/) and then imported the results into Cytoscape software (http://www.cytoscape.org/, version 3.8.2) to further analyze functional PPI networks. EVenn (http://www.ehbio.com/test/Venn/#/) was used to create Venn diagrams.

### Cell transfection with siRNAs

SiRNA against intracellular adhesion molecule-1 (ICAM-1) (siICAM-1) was provided by GenePharma (Shanghai, China) and transfected into TMCs using Lipofectamine 2000 reagent after induction with H_2_O_2_ for 24 h. The sense strand of siICAM-1 used for gene knockdown was as follows: 5ʹ-GCCAACCAAUGUGCU AUUCAAdTdT-3ʹ. The scrambled siRNA sequence was UUCUCCGAACGUGUCACGUdTdT. Before transfection, the TMCs were cultured in 6‐well plates with complete medium for 24 h. siICAM-1 was transfected with Lipofectamine 2000 (Thermo) in serum‐free DMEM for 6 h, and then, the mixture was replaced with complete medium. Western blotting was used to verify protein knockdown 24 h post-transfection.

### Western blotting

TMCs were lysed with lysis buffer (20 mmol/L HEPES, 150 mmol/L NaCl, 1 mmol/L EGTA, 1 mmol/L EDTA, 10% glycerol, 1 mmol/L MgCl_2_, 1% Triton X‐100). The extracts were centrifuged at 12,000 rpm for 20 min at 4 °C, and the supernatants were collected. The protein extracts were separated on 10% polyacrylamide‐SDS gels, transferred to PVDF membranes and then blocked with 5% skimmed milk powder for 1 h. After incubation with primary antibodies (anti-ICAM, Abcam) overnight, the membranes were washed three times and incubated with fluorescent secondary antibodies at room temperature for 2 h. The fluorescent signals were captured using an infrared imager (Millipore, USA).

### Cell viability assay

TMC viability was examined using a CCK-8 (Dojindo Molecular Technologies, Gaithersburg, MD, USA). TMCs were seeded in 96-well plates and incubated at 37 °C for 24 h. After cell transfection and/or stimulation with H_2_O_2_, the culture medium was replaced with TMC medium (TMCM) containing 10% CCK-8 solution, and the cells were incubated at 37 °C for an additional 2 h. Finally, the absorbance at 450 nm was detected by using a microplate reader (Bio-Rad, Hercules, CA, USA).

### Statistical analysis

The two-tailed Student t test was used for statistical analysis. All data are expressed as the mean ± SE, and a *P* value < 0.05 was considered to indicate statistical significance.

## Results

### Cell identification and model establishment

**Fig. 1:**
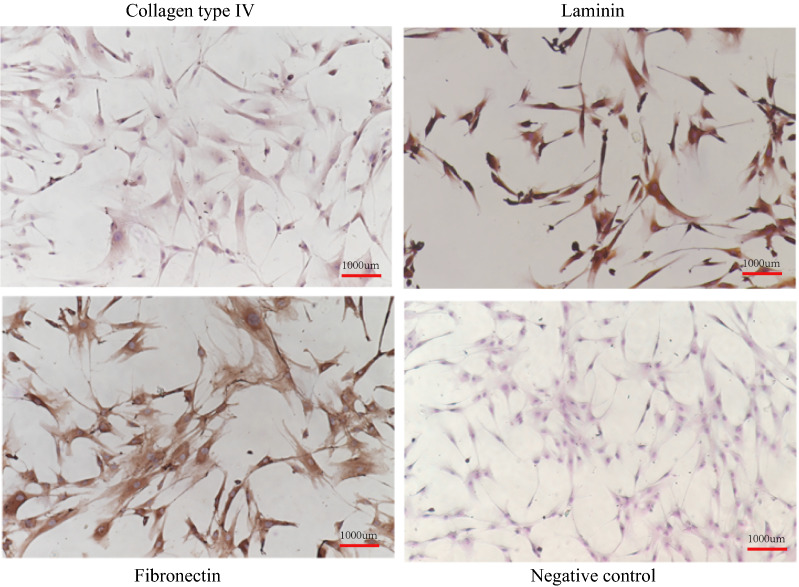
The identification of TM cells. The Collagen Type IV, Laminin and Fibronectin protein were all fully expressed after immunocytochemistry

**Fig. 2:**
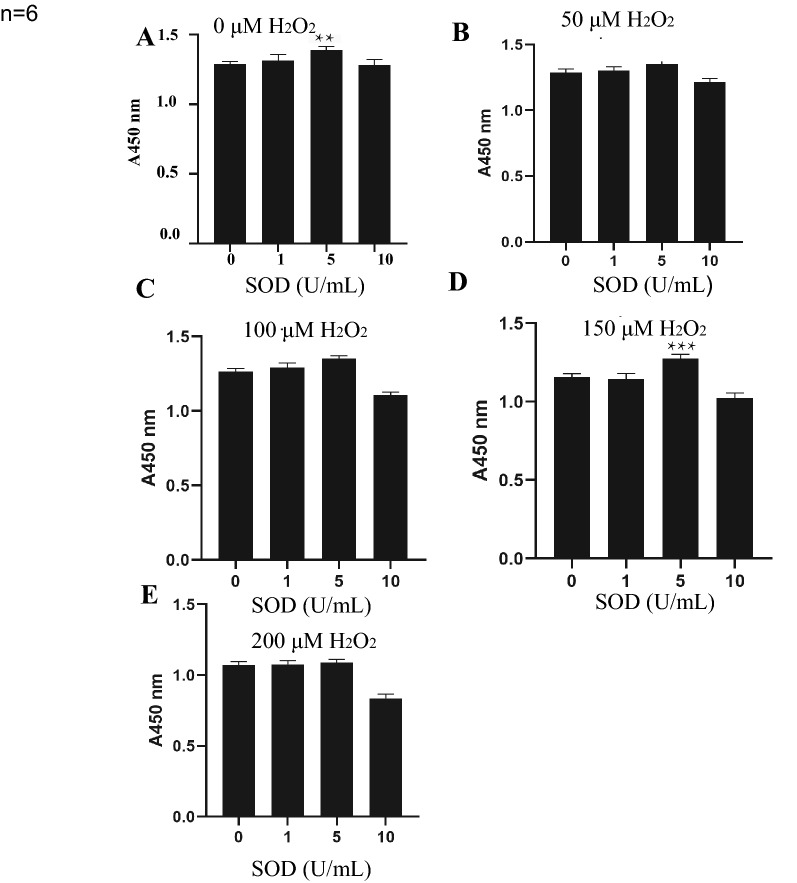
The result of CCK8 under different concentrations of H_2_O_2_ and SOD. The abscissa is the concentration of SOD and the ordinate is the OD value. “*” represent the *P* value < 0.05, “**” represent the *P* value < 0.01 “***” represent* P* value< 0.001

The immunocytochemistry results for cells with collagen type IV, laminin and fibronectin antigens are shown in Fig. 1. We observed that these antigens were all expressed in the cells, which confirmed that the cells we cultured were TMCs. The CCK-8 results are also shown in Fig. 2. When the concentration of H_2_O_2_ was 150 μM and the concentration of SOD was increased to 5 U/mL, the numbers of cells significantly differed between the H_2_O_2_-treated group and the SOD-pretreated group. Hence, we determined that the H_2_O_2_-treated group (group H) represented TMCs cultured with 150 μM H_2_O_2_ for 24 h, and the SOD-pretreated group (group S) represented TMCs pretreated with 5 U/mL SOD for 30 min and then subjected to the same conditions as the H_2_O_2_-treated group.

### LC–MS/MS analysis and identification of DEPs

**Fig. 3:**
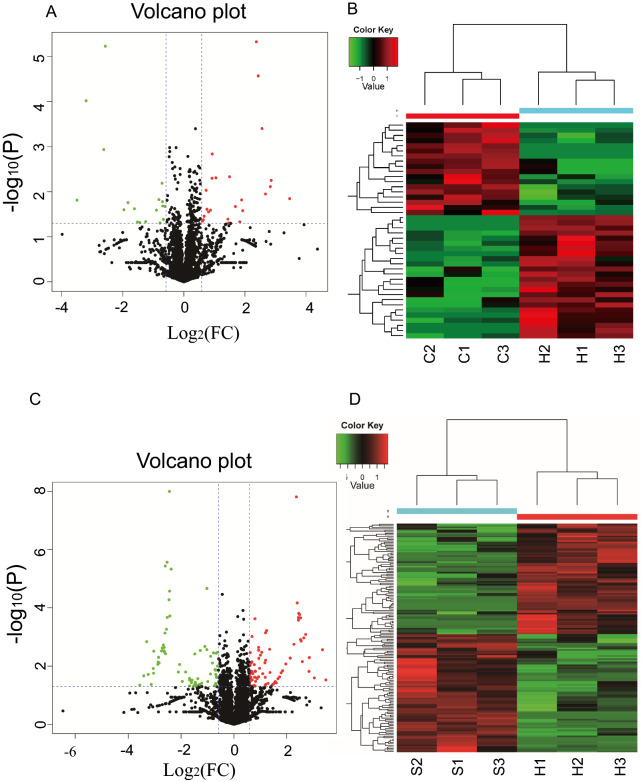
Volcano plot and heatmap of differentially expressed proteins in TM cells. **A**, **B** Are DEPs between the group H and the group C while **C** and **D** are differential protein expression between the group S and the group H. In Volcano plot (**A** and **C**), the abscissa is the FC in protein concentration and the ordinate is the statistical significance. Red and green dots presents proteins with significant differences (green, FC) ≥ 1.5 and *P* value < 0.05 (up) and red, FC ≤ 0.667 and P < 0.05(down)); black dots are proteins without significant change. In heatmap (**B** and **D**), each row is a protein, each column is a sample/repeat, and different color represents different quantity of expression

Each group included three samples. After LC–MS/MS analysis, we identified 24 upregulated proteins and 18 downregulated proteins between group H and group C (Fig. 3A, B), and we identified 78 upregulated proteins and 73 downregulated proteins between group S and group H (Fig. 3C, D).

### GO functional annotation and enrichment analysis

**Fig. 4:**
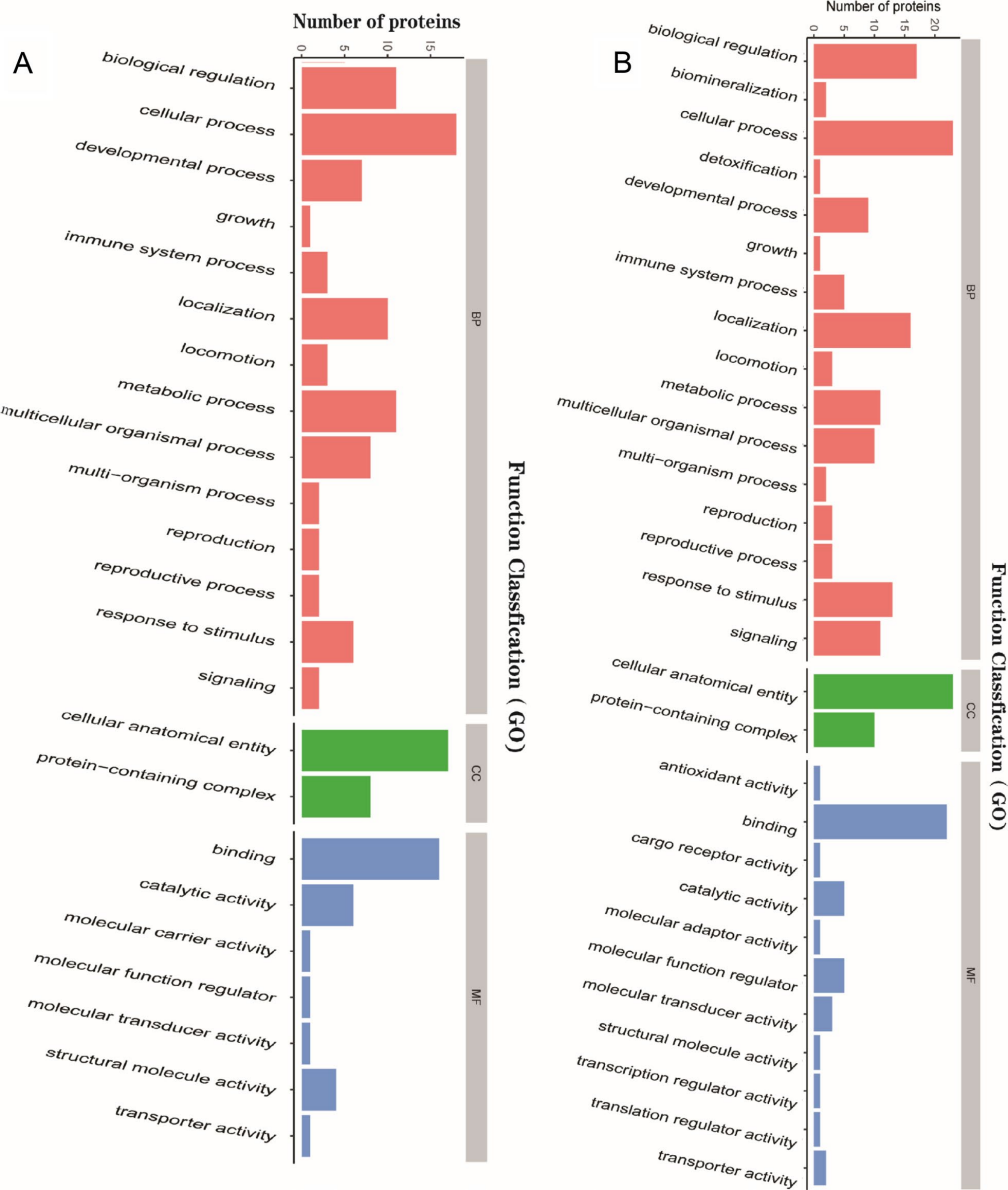

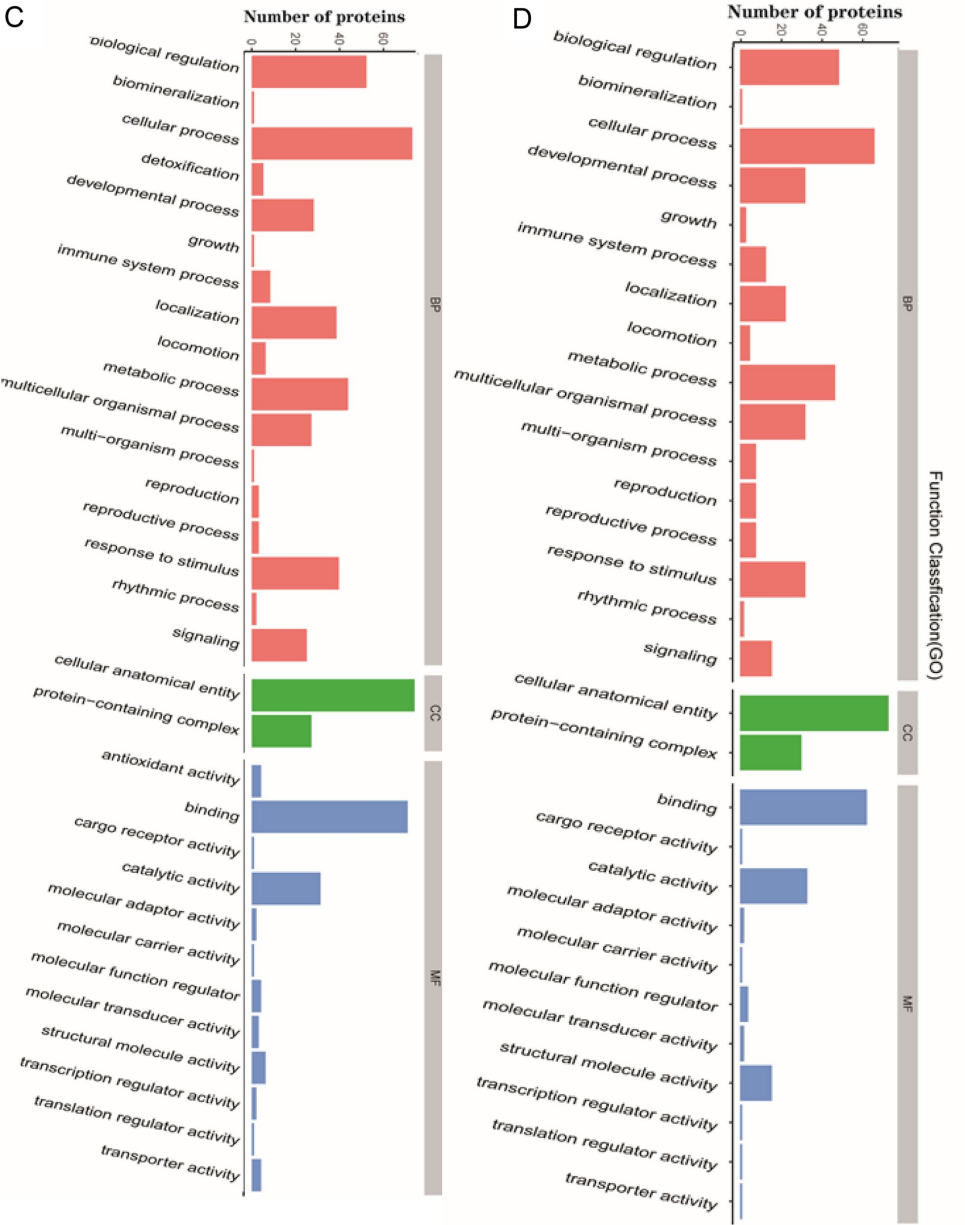
Gene ontology (GO) annotation for functional classification. **A** and **B** were the result of upregulated (**A**) and downregulated (**B**) DEPs between group H and group C, while **C** and **D** were the result of that between group S and group H. The abscissa in the graph represents the enriched GO functional classification, the ordinate represents the number of differentiated proteins under each functional classification. Different color represents different categories

**Fig. 5:**
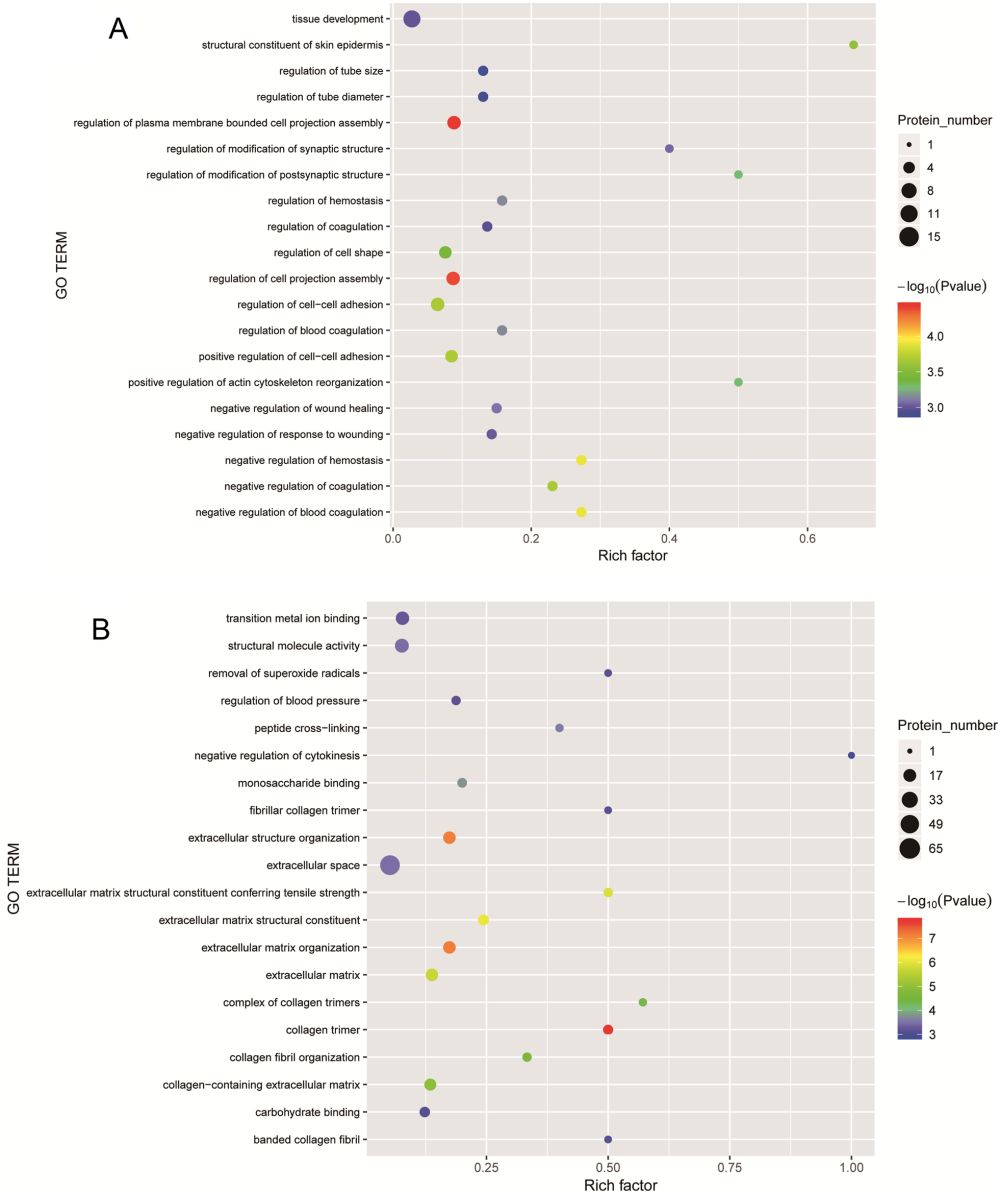
GO function enrichment analysis for all DEPs between groups. **A** The result between group H and group C, while **B** is that between group S and group H. The abscissa represents the enrichment factor. The ordinate are the GO term description. Bubble size represents the number of DEPs in GO classification; the enrichment test* P* value obtained by using Fisher exact test; − log10(*P* value): the logarithmic conversion of Fisher exact test *P *value, different color represents different *P* value

GO functional annotation was performed based on three categories: BP, MF and CC (Fig. 4). We found that between group H and group C (Fig. 4A and B), the major GO terms were related to “cellular process” (up: n = 23, down: n = 18) and “biological regulation” (up: n = 17, down: n = 11) in the BP category, “cellular anatomical entity” (up: n = 23, down: n = 17) in the CC category and “binding” (up: n = 22, down: n = 16) in the MF category. Furthermore, between group S and group H (Fig. 4C and D), the major GO terms were the same as those between group H and group C: “cellular process” (up: n = 68, down: n = 70) and “biological regulation” (up: n = 50, down: n = 50) for the BP category, “cellular anatomical entity” (up: n = 75, down: n = 71) for the CC category and “binding” (up: n = 64, down: n = 68) for the MF category. In addition, the results of GO functional enrichment analysis are shown in Fig. 5. The enriched DEPs between group H and group C (Fig. 5A) were mainly related to the functional terms “regulation of plasma membrane-bounded cell projection assembly”, “negative regulation of blood coagulation” and “regulation of cell projection assembly”, whereas the enriched DEPs between group S and group H (Fig. 5B) were related primarily to the functional terms “collagen trimer”, “extracellular matrix organization” and “extracellular structure organization”.

### PPI analysis and Venn analysis

**Fig. 6:**
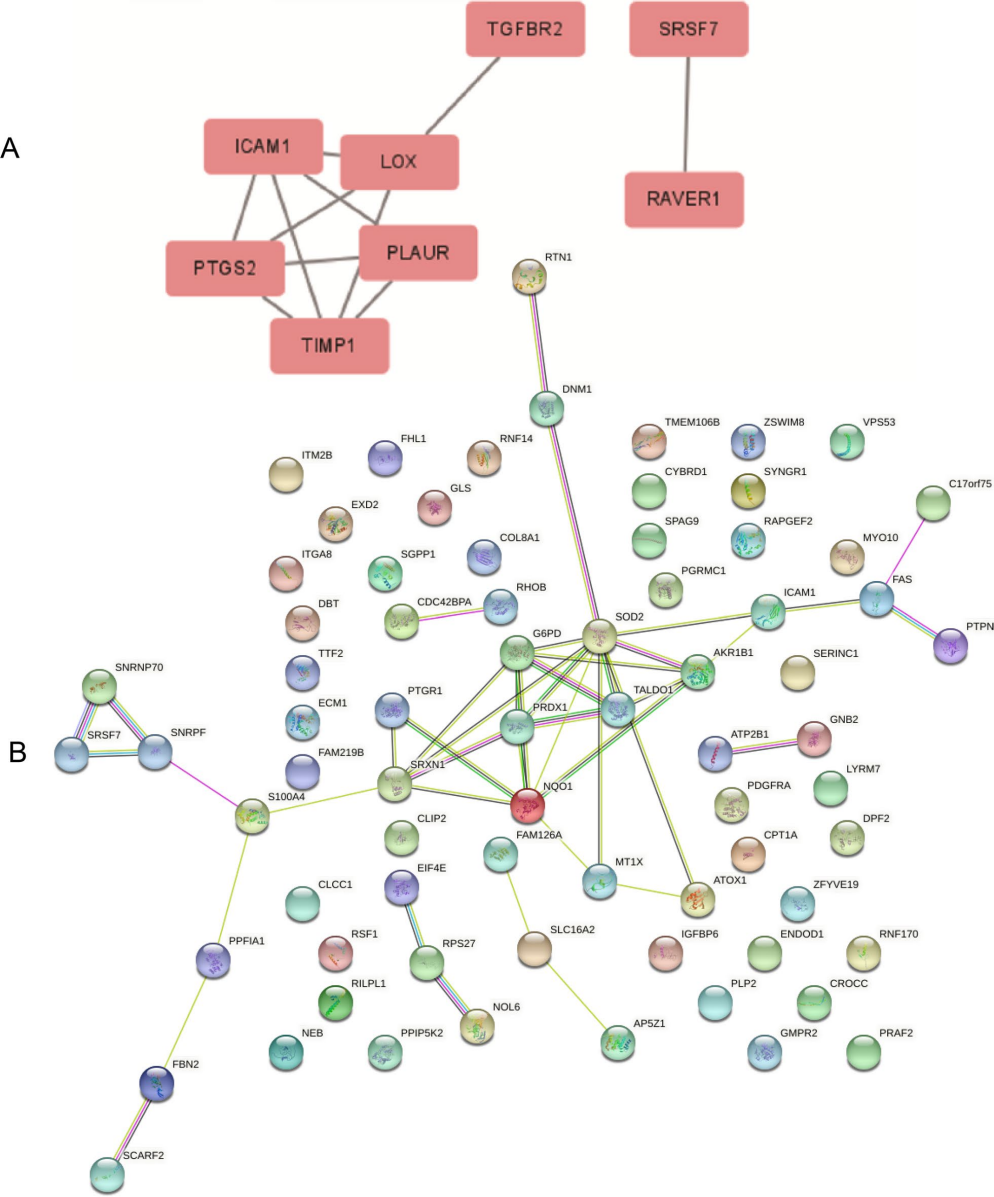
The relatively concentrated nets were obtained by PPI analysis. **A** Represents the key target network for upregulated DEPs in group H and group C. **B** Represents the key target network for downregulated DEPs in group S and group H, the line represents the protein interaction recorded or predicted by STRING, each box represents the key proteins recorded by CytoScape

**Fig. 7:**
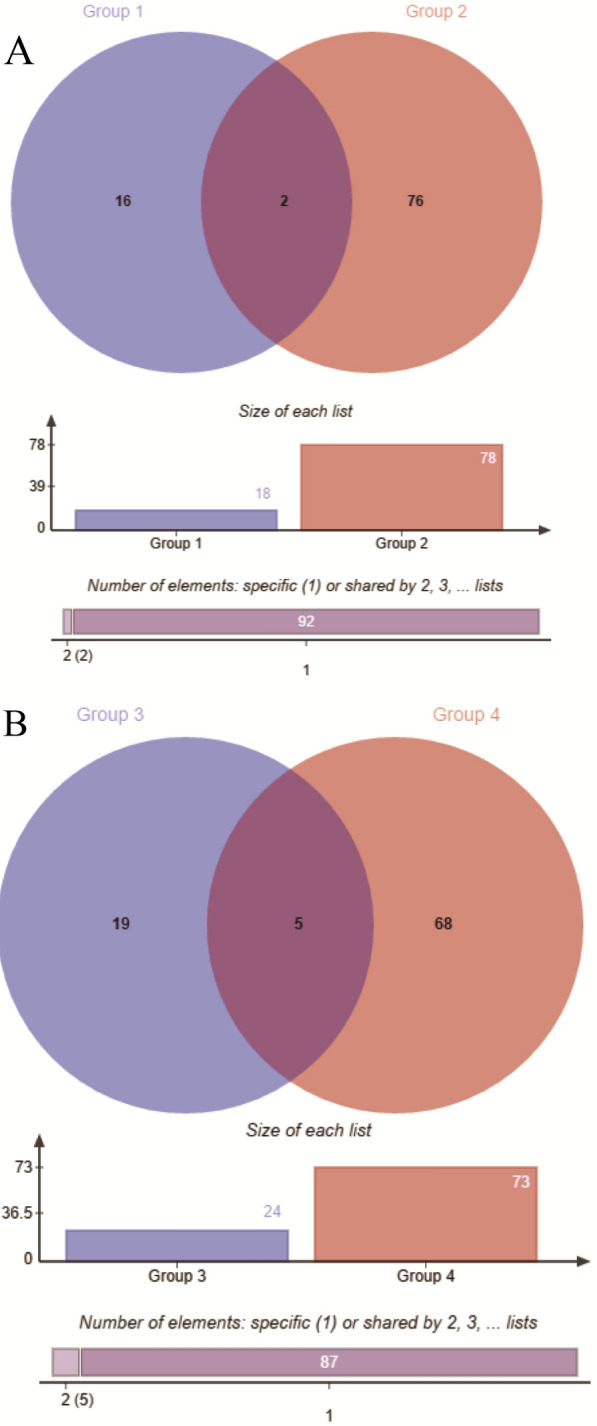
The venn diagram of the DEPs between group H and group C vs group S and group H. **A** Represents the common DEPs decreased between group H and group C while increased between group S and group H. **B** Is contrary to **A**

To determine the molecular mechanisms by which OS and antioxidant stress occur in TMCs, we used STRING software combined with Cytoscape software to analyze the DEPs in the different groups. Figure 6A shows the key PPI network for the upregulated DEPs between group H and group C. Figure 6B shows the key PPI network for the downregulated DEPs between group S and group H. Furthermore, to determine the mechanism by which SOD protects TMCs, we conducted Venn analyses to identify the key proteins (Fig. 7).

### KEGG pathway analysis for DEPs

**Fig. 8:**
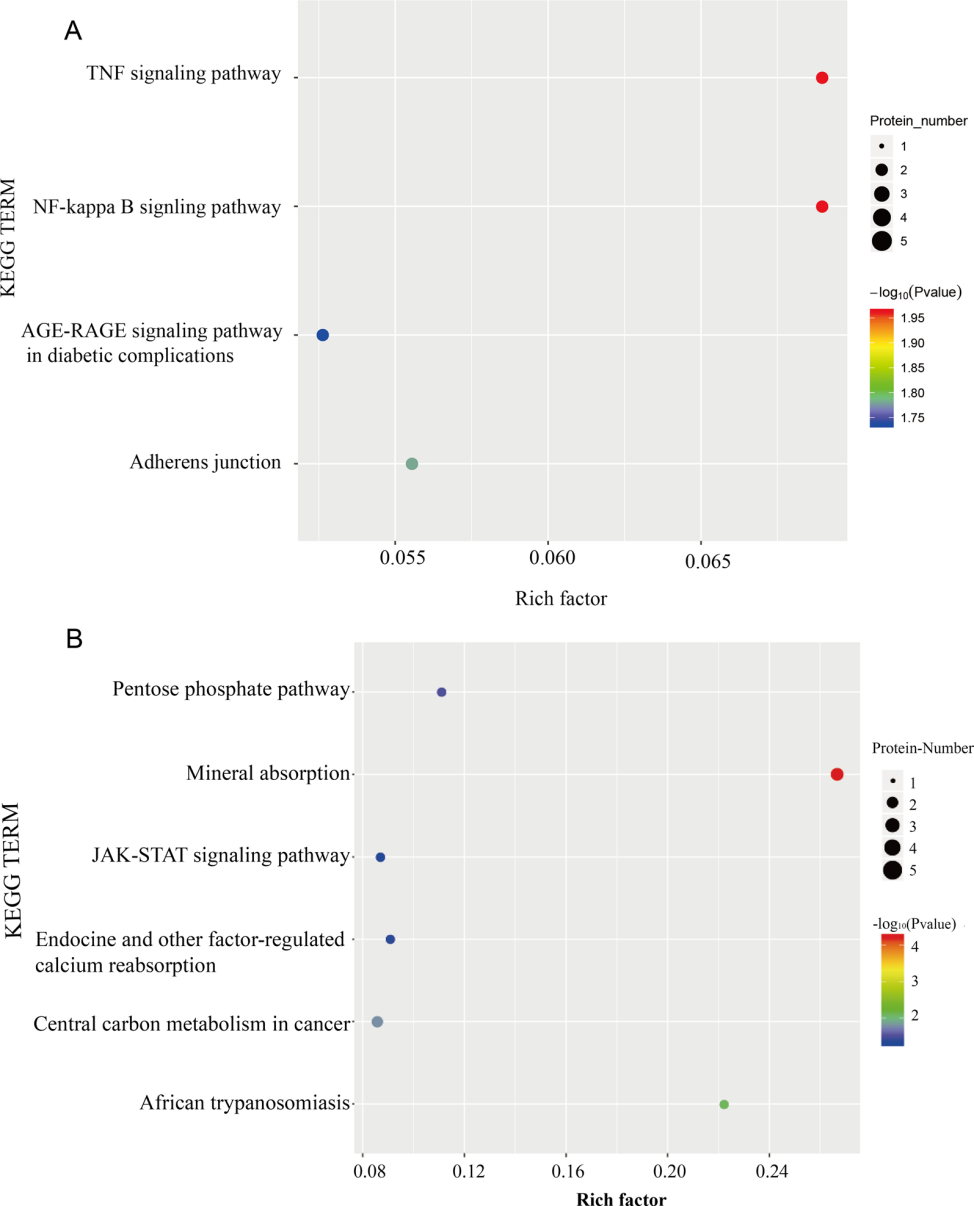
Kyoto Encyclopedia of Genes and Genomes (KEGG) pathway enrichment of DEPs. **A** Represents the result of up DEPs between group H and group C, **B** Represents the result of down DEPs between group S and group H. The ordinate lists the KEGG term description. Bubble size represents the number of DEPs in the KEGG pathway; Fisher exact test *P* value: the enrichment test *P* value obtained by using Fisher exact test and expressed by − log10 (*P* value)

**Fig. 9:**
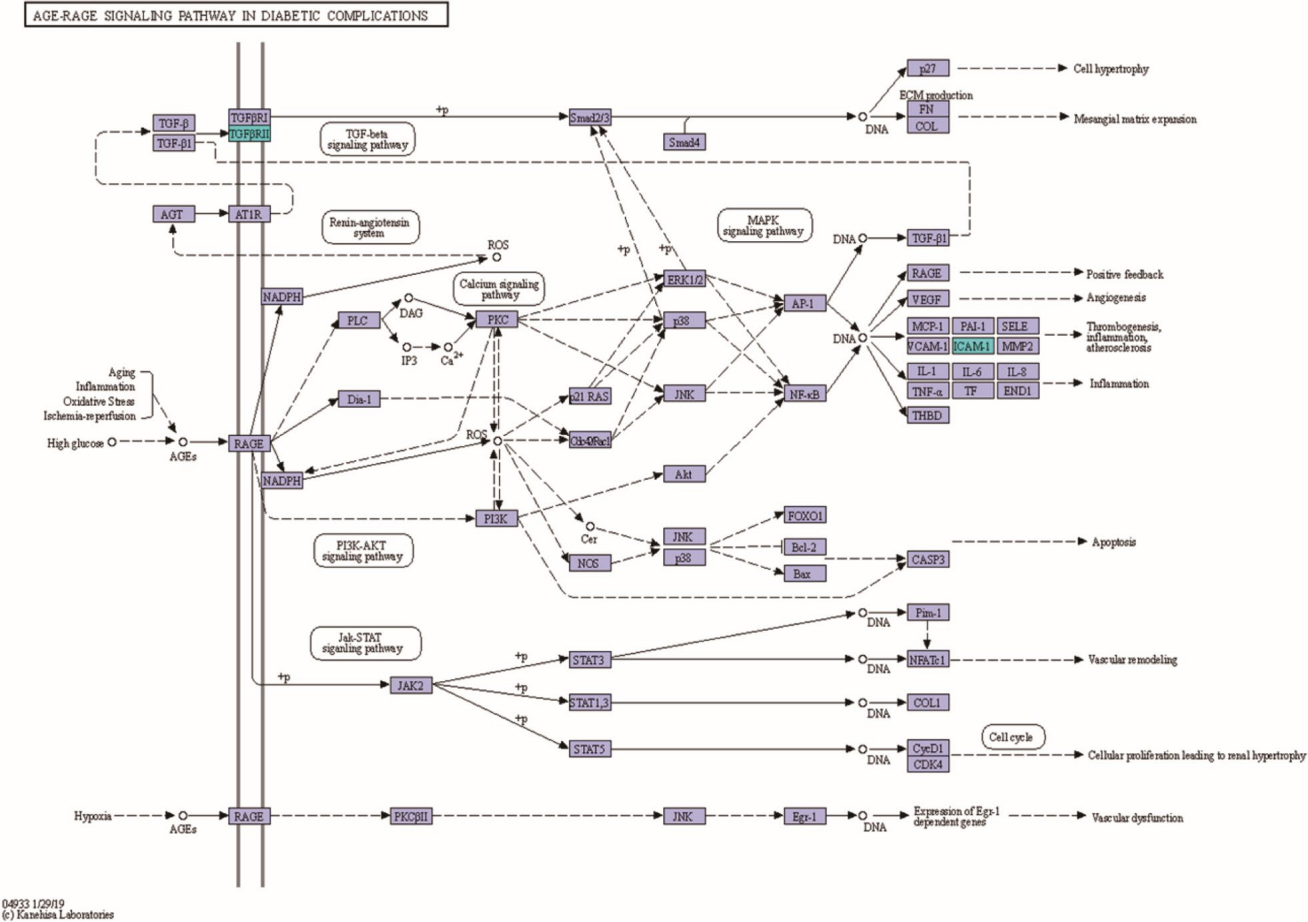
The process and major proteins of AGE-RAGE signaling pathway in diabetic complications. The upregulated DEPs, ICAM1 and TGFβr2 were highlighted in a blue frame

**Fig. 10:**
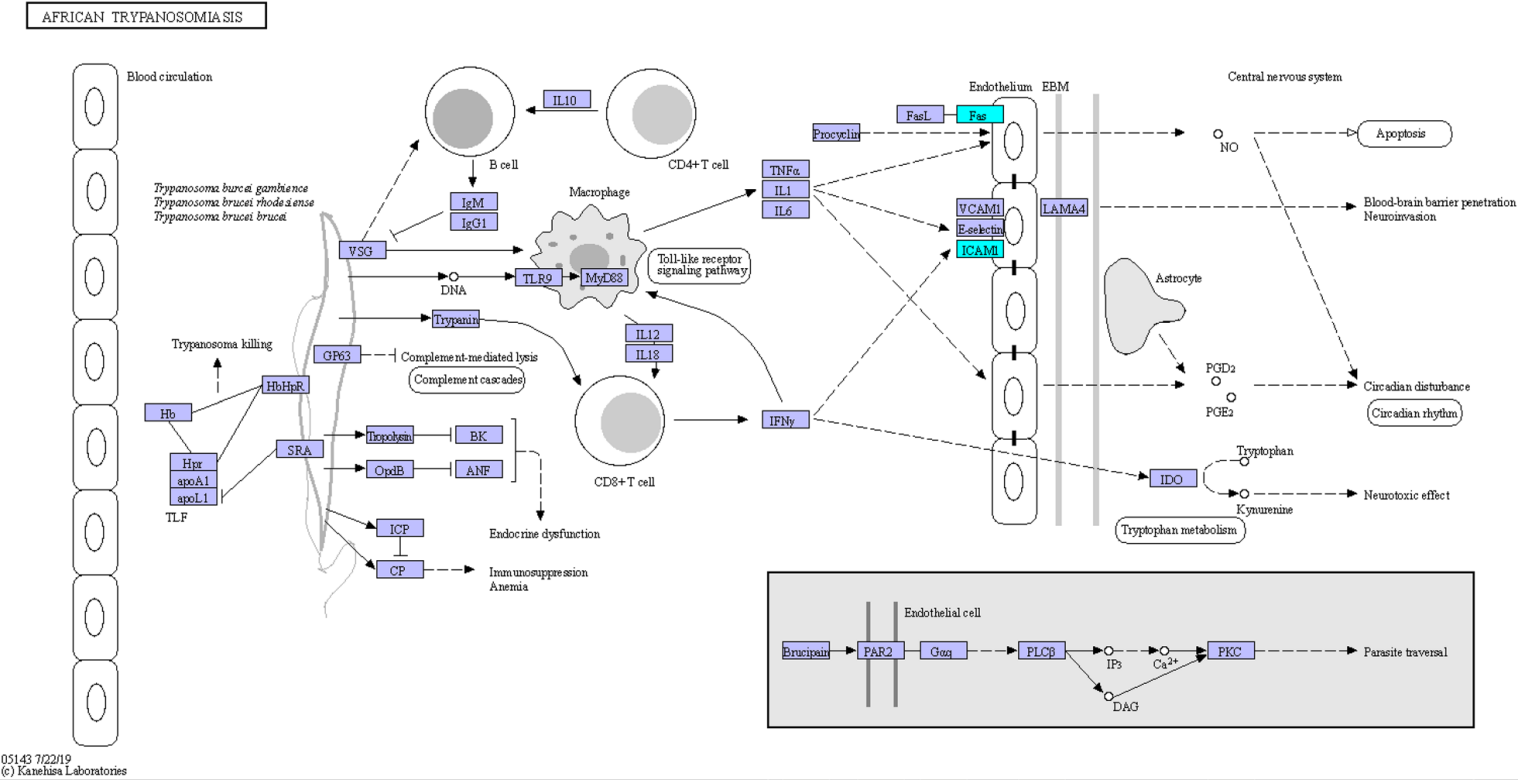
The process and major proteins of African trypanosomiasis. The downregulated DEPs, ICAM-1 was highlighted in a blue frame

We conducted KEGG signaling pathway analysis for DEPs. The results of KEGG pathway enrichment between group H and group C are shown in Fig. 8A. The top four pathways in KEGG enrichment were the TNF signaling pathway [major proteins: ICAM-1 and prostaglandin-endoperoxide synthase 2 (PTGS2)], the NF-kB signaling pathway (major proteins: ICAM-1 and PTGS2), adherens junctions (major proteins: BAIAP2 and TGFβr2) and the AGE-RAGE signaling pathway in diabetic complications (major proteins: ICAM-1 and TGFβr2). We deemed the AGE-RAGE signaling pathway in diabetic complications noteworthy after our KEGG pathway annotation and enrichment analyses (Fig. 9). The results of KEGG pathway analysis for the downregulated DEPs between group S and group H are shown in Fig. 8B. The top three pathways were mineral absorption (major proteins: ATOX1 and CYBRD1), African trypanosomiasis (major proteins: ICAM1 and FAS), shown in Fig. 10, and central carbon metabolism in cancer (major proteins: GLS and G6PD).

### ICAM-1 knockdown rescues the viability of TMCs treated with H_2_O_2_

**Fig. 11:**
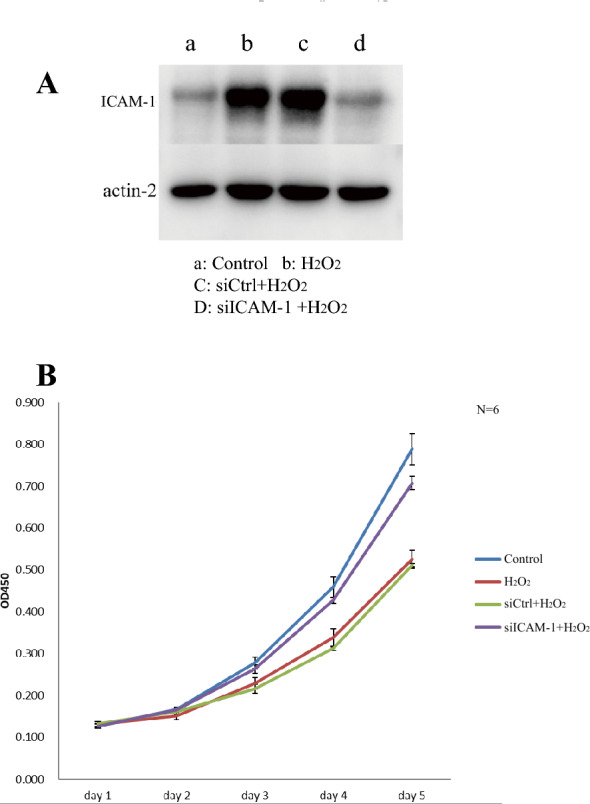
The expression of ICAM-1 and the viability of TM cells in different groups. **A** The expression of ICAM-1 after transfection of siICAM-1. **B** The viability of TM cells with different condition over time. The abscissa is the time and the ordinate is the OD value which represents the viability of TM cells

To further study the regulation of ICAM-1 in H_2_O_2_-treated TMCs, we transfected siRNA into TMCs and used a CCK-8 assay to test TMC viability. The expression of ICAM-1 in the H_2_O_2_-treated group was higher than that in the blank group, and the expression in the group transfected with siICAM-1 was lower than that in the H_2_O_2_-treated group (Fig. 11A). Moreover, the viability of TMCs in the different groups was obviously different over time. After treatment with H_2_O_2_, viability was reduced, while viability recovered after transfection with siICAM-1 compared to the H_2_O_2_-treated group (Fig. 11B).

## Discussion

Glaucoma is a chronic progressive optic neuropathy characterized by irreversible damage to the retinal nerve fiber layer (RNFL) as well as peripheral and occasional central vision loss. Elevated IOP is the major risk factor for glaucoma; hence, lowering IOP is regarded as the first-line treatment. However, the efficacy of IOP reduction is undoubtedly insufficient; furthermore, normal-tension glaucoma exists. Sacca et al. [3] reported that OS in TMCs affects gene and protein expression, contributing to the development of glaucoma. In our study, we cultured TMCs and compared the differentially expressed proteins through label-free LC–MS/MS proteomics analyses to identify the key proteins and pathways in cells under OS and antioxidant stress. Finally, a list of key proteins was identified, and the major pathways were explored.

Figure 1 shows the results of cell identification. The expression of collagen type IV, laminin and fibronectin demonstrated that the cells we cultured were TMCs. As shown in Fig. 2, we found that apoptosis increased as the H_2_O_2_ concentration increased. SOD had a protective effect on TMCs; however, when the concentration of H_2_O_2_ reached 200 μM, the apoptosis of TMCs increased. Moreover, when the concentration of SOD reached 10 U/mL, SOD had a harmful effect on TMCs. We determined that the difference was most significant when the concentration of H_2_O_2_ was 150 μM and the concentration of SOD was 5 U/mL. Hence, we selected these concentrations for further experiments.

Our study included three groups: group C, group H and group S. We used label-free LC–MS/MS proteomics analyses to analyze the DEPs. After comparing group H and group C, we obtained 24 upregulated proteins and 18 downregulated proteins, as shown in Fig. 3A and B. We obtained 78 upregulated proteins and 73 downregulated proteins between group S and group H. After GO, KEGG and PPI analyses, we ultimately obtained a list of key proteins and pathways that play important roles in the molecular mechanisms in TMCs under OS and antioxidant stress.

H_2_O_2_ is often used to induce OS. In our study, we cultured TMCs with 150 μM H_2_O_2_ for 24 h. Among the upregulated proteins, PPI analyses revealed PTGS2, TGFβr2 and ICAM-1 as important DEPs between group H and group C. Identification of these proteins suggests the mechanism by which OS damages TMCs.

The levels of PTGS2 (also known as COX2), a rate-limiting enzyme in the conversion of arachidonic acid into prostaglandins, often increase during inflammation. In a study by Li et al. [13], melanocytes were treated with H_2_O_2_, RNA was extracted, and the differential expression profiles of RNAs were detected and analyzed through GO and KEGG analyses. Li et al. also concluded that PTGS2 might play central regulatory roles in the OS response. OS often damages cells by increasing apoptosis and inflammation. PTGS2 is a key enzyme involved in inflammation that can activate the PI3-K/AKT and PKA/CREB pathways, which facilitate apoptosis and inflammation [14]. Furthermore, PTGS2 is an NF-kB target gene whose promoter has been shown to contain several binding sequences for the transcription factor NF-ĸB [15]. The NF-ĸB pathway can activate the expression of PTGS2, and a growing body of evidence suggests that the NF-ĸB pathway participates in inflammatory and immune responses, activating the expression of adhesion molecules, cytokine receptors, and numerous cytokines, which effectively affects the process of apoptosis [16]^.^ In our study, the NF-ĸB pathway was identified as the major KEGG pathway in the upregulated DEPs between group H and group C. It can be concluded that NF-ĸB stimulates PTGS2 expression and may be largely responsible for the damage to TMCs after treatment with H_2_O_2_.

Transforming growth factor β (TGF-β) exists in three isoforms, TGFβ1, TGFβ2, and TGFβ3. Numerous studies have demonstrated that TGF-β increases ROS production and suppresses the antioxidant system, thereby inducing OS. It can activate cells to produce ROS via NADPH oxidase [17], leading to apoptosis. Active TGF-β binding to TGFβ2 on the cell membrane activates TGFβR1, initiating the TGFβ signaling pathway and regulating cell hypertension, proliferation, apoptosis, differentiation, and morphogenesis. The TGF-β/Smad signaling pathway is an important downstream signal transduction pathway of TGF-β-mediated apoptosis. There are eight Smad proteins, and Smad2 and Smad3 belong to the receptor-regulated Smad (R-Smad) family. When TGFβ1 is activated, heterogeneous complexes are formed; Smad2 and Smad3 are then phosphorylated and can bind with Smad4 (the comediator Smad, Co-Smad) [18]. Smad complexes are translocated into the nucleus, where they regulate the transcription of target genes and the production of ECM proteins [18], which has a negative effect on cell growth. Furthermore, TGF-β plays a pivotal role in the development of fibrosis [19]. Zhang et al. [20] used DZ2002 to suppress the TGF-β/Smad signaling pathway to decrease fibrosis in human dermal fibroblasts under conditions of systemic sclerosis. In our study, TGFβr2 was increasingly expressed in TMCs, and the TGF-β/Smad signaling pathway was involved in the AGE-RAGE signaling pathway in diabetic complications (Fig. 8), leading to thrombogenesis, inflammation and atherosclerosis. We can conclude that the TGF-β/Smad signaling pathway may enhance apoptosis of TMCs or induce fibrosis in TMCs to affect TMC function. One of the most important proteins involved in the TGF-β/Smad signaling pathway is ICAM-1. ICAM-1, a member of the immunoglobulin superfamily, is often expressed at low levels by endothelial cells and is highly expressed and induced by a variety of inflammatory cytokines when OS occurs. In endothelial cells, ICAM-1 often plays a key role in mediating firm adhesion of leukocytes and participates in many physiological processes [21]. It has been reported that ICAM-1 can regulate endothelial cell permeability in healthy and inflamed tissue [22, 23]. When ICAM-1 levels increase, JNK is activated, leading to internalization of VE-cadherin, disruption of cell junctions and impairment of cell barrier function [21]. In addition, ICAM-1 can activate monocytes to enhance the stimulation and transmigration of inflammatory cells into the ECM [24, 25], substantially increasing apoptosis. In our study, ICAM-1 levels were significantly increased, and ICAM-1 was the major protein in the TNF signaling pathway, NF-kB signaling pathway and AGE-RAGE signaling pathway in diabetic complications. These pathways were all major KEGG pathways in TMCs under OS that were demonstrated to be obviously related to apoptosis. In summary, ICAM-1 levels increased when OS occurred in TMCs in our study, and ICAM-1 participated in the PTGS2/NF-ĸb pathway, TGF-β/Smad signaling pathway and AGE-RAGE signaling pathway in diabetic complications to reduce the viability of TMCs.

SOD is the first-line antioxidant and can effectively eliminate ROS. Jiang et al. [26] demonstrated that adeno-associated virus (AAV)-mediated pretreatment with SOD2 is able to attenuate OS. In our study, we pretreated TMCs with SOD for 30 min and then exposed the TMCs to 150 μM H_2_O_2_. After conducting Venn analyses, we found that ICAM-1 was also the key DEP whose expression was downregulated after SOD was added to TMCs. As discussed previously, increased ICAM-1 is one of the most important factors that damages TMCs under OS. Moreover, ICAM-1 is also the key protein involved in African trypanosomiasis, one of the major KEGG pathways for downregulated DEPs between group S and group H. Hence, we can conclude that SOD may suppress the OS induced by H_2_O_2_ by decreasing ICAM-1 levels to reduce the apoptosis of TMCs.

To further study the function of ICAM-1, we reduced the expression of ICAM-1 via transfection of siICAM-1. Western blotting was used to test the expression of ICAM-1, and a CCK-8 assay was used to measure the viability of TMCs. We found that after treatment with H2O2, the expression of ICAM-1 in TMCs was elevated, while transfection with siICAM-1 inhibited ICAM-1 expression and increased viability over time. All the results illustrate that oxidative injury of human TMCs induced by H_2_O_2_ is related to ICAM-1 expression and that knockdown of ICAM-1 attenuates the injury induced by H_2_O_2_.

## Conclusion

In this study, we used label-free LC–MS/MS proteomics analyses to analyze the proteomic changes that occurred when TMCs were subjected to OS induced by H_2_O_2_ and antioxidant stress induced by SOD. The results of GO analysis, KEGG analysis and PPI network analysis indicated that the upregulated proteins PTGS2, TGFβr2 and ICAM-1 were the key proteins that participate in the PTGs2/NF-ĸb pathway, TGF-β/Smad signaling pathway and AGE-RAGE signaling pathway in diabetic complications, leading to apoptosis and fibrosis of TMCs. SOD may protect TMCs mainly by decreasing the levels of ICAM-1, which participates in African trypanosomiasis inhibiting apoptosis. Notably, higher expression of ICAM-1 was associated with lower viability of TMCs. These data provide valuable insights into the roles of these key proteins and pathways, which may be regarded as new therapeutic targets for glaucoma.

## Data Availability

Not applicable.

## Abbreviations

TM: Trabecular meshwork
HTMC: Human trabecular meshwork cell
IOP: Intraocular pressure
LC–MS/MS: Liquid chromatography–tandem mass spectrometry
SOD: Superoxide dismutase
PTGS2: Prostaglandin-endoperoxide synthase
TGFβr2: Transforming growth factor β receptor 2
ICAM-1: Intercellular adhesion molecule 1
OS: Oxidative stress
ROS: Reactive oxygen species
ECM: Extracellular matrix
DEP: Differentially expressed protein
